# Mechanical power and short-term mortality in critically ill patients with ARDS on mechanical ventilation: Insights from the MIMIC-IV database

**DOI:** 10.1371/journal.pone.0341923

**Published:** 2026-02-02

**Authors:** Zhenyan Lu, Chunqiao He, Qidong Jiang, Mengyuan Wang, Wei He, Shuang Wang, Ying Xie, Zhao Peng, Xue Pan

**Affiliations:** 1 Medical Equipment Department, The Affiliated Hospital of Southwest Medical University, Luzhou, Sichuan, China; 2 Intensive Care Unit, The Affiliated Hospital of Southwest Medical University, Luzhou, Sichuan, China; 3 Big Data Research Center, University of Electronic Science and Technology of China, Chengdu, Sichuan, China; Centre Hospitalier Universitaire Farhat Hached de Sousse, TUNISIA

## Abstract

Mechanical power (MP), or an integrated measure of ventilatory stress, has been proposed as an important determinant of ventilator-induced lung injury and clinical outcomes. However, its prognostic significance in patients with acute respiratory distress syndrome (ARDS) remains incompletely understood. We selected adult patients with Berlin ARDS from MIMIC-IV v3.1. Cox proportional hazard regression models were built to explore the relationship between MP quartiles and in-hospital, 28-day, and 90-day mortality, with progressive adjustment for demographics, severity scores, and ventilatory parameters. We also explored model discrimination and calibration, conducted comparative receiver-operating characteristic curves (ROC) and restricted cubic spline (RCS) analyses, and performed Kaplan-Meier, subgroup, and sensitivity analyses as complementary assessments. A total of 1878 patients were included. Each increase in MP was significantly associated with higher in-hospital, 28-, and 90-day mortality. Using full models, Q4 had significantly higher risk than Q1 (HR 1.32; 95% CI 1.06–1.64). MP had stable discriminative performance with good calibration. Using ROC, MP had significantly better performance than traditional predictors. Models built with RCS showed no evidence for curvature. Survival analysis showed successively decreased probabilities with increasing quarters from Q1 to Q4. Sensitivity analyses were largely consistent, with subgroup analyses supporting findings. In patients with ARDS, elevated MP was independently associated with in-hospital, 28-day, and 90-day mortality. These findings suggest that MP may serve as an integrated prognostic marker of ventilatory load rather than a standalone target. Our results warrant further prospective research to determine whether incorporating MP into lung-protective strategies can improve clinical outcomes.

## Introduction

Acute respiratory distress syndrome (ARDS) remains an ongoing source of morbidity and mortality for critically ill subjects, in spite of many years of improvements in supportive care. One major factor in outcomes for subjects with ARDS is ventilator-induced lung injury (VILI). It is caused by excessive forces being applied to an injured lung [1–4]. Mainstays in traditional ventilation strategies, such as tidal volume (Vt), plateau pressure (Pplat), and driving pressure (ΔP), have long-standing relationships with outcomes such as mortality, with these elements forming the cornerstone for lung-protective ventilator strategies. Of note, ΔP has been suggested to represent a powerful predictor for survival, likely having predictive value for outcomes exceeding those for Vt or Pplat [5–8].

Mechanical power (MP) has also recently been defined to represent the overall energy transferred from the ventilator to the lung in each unit of time. MP is postulated to provide an overall measurement incorporating Vt, respiratory rate (RR), peak pressure (Ppeak), and positive end-expiratory pressure (PEEP), addressing changes in overall mechanical loading not captured with other ventilator variables [9,10]. Research studies have indicated increased MP to worsen VILI outcomes, yet whether MP gives additional information for predictive analysis regarding ΔP or lung compliance remains controversial. Previous studies have included small sample sizes, had varied subject definitions, or inadequate adjustments for severity, with no studies examining dose-response relationships [11–13].

For dealing with these uncertainties, we employed the big MIMIC-IV database to analyze the relationship between MP and short-term mortality in patients with ARDS. We explored mortality rates for different quartiles of MP, checked for presence of nonlinearity, and applied multiple tests to check for robustness.

## Method

### Data source

**Fig 1:**
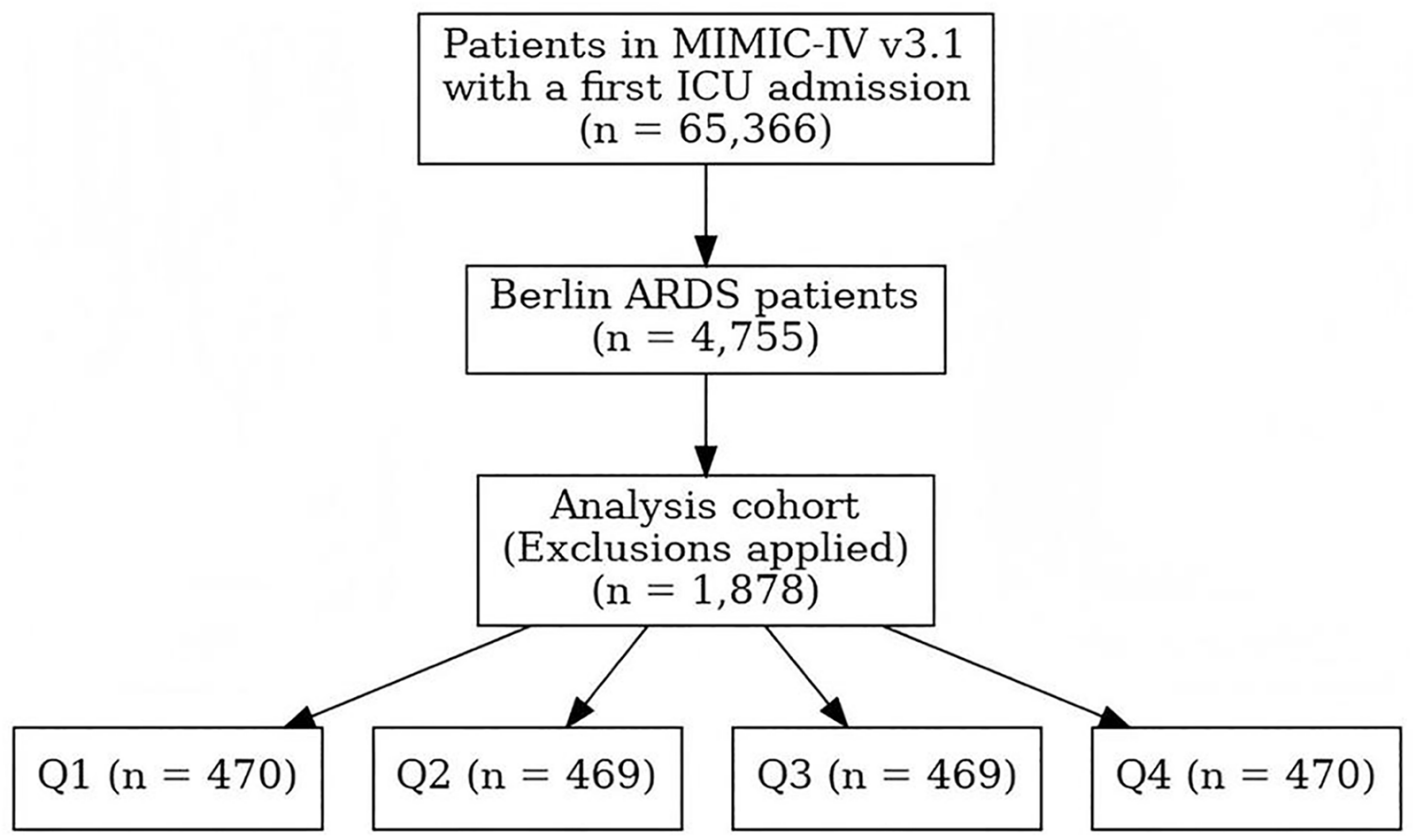
Flowchart of patient selection for the study.

We identified patients with ARDS using an operationalization of the Berlin definition in the MIMIC-IV version 3.1 database, which is a collection of data gathered from intensive care unit (ICU) patients from Beth Israel Deaconess Medical Center in Boston, Massachusetts. We restricted the cohort to adult ICU admissions with evidence of invasive mechanical ventilation. Within each ICU stay, we searched the first 7 days after ICU admission for arterial blood gas measurements and calculated the PaO₂/FiO₂ (P/F) ratio. ARDS was considered present if at least one arterial blood gas during invasive mechanical ventilation demonstrated a P/F ratio ≤300 mmHg [1]. Exclusion criteria were: 1) Patients aged less than 18 years; 2) Patients who were not ventilated mechanically throughout their intensive care stay; 3) Patients with missing critical variables that did not allow for calculation of MP (Fig 1). Author Zhao Peng accessed databases and extracted data after completing necessary institutional training (Record ID: 71678163). Investigators with valid accreditation may access publicly available databases including MIMIC-IV. Additionally, the MIMIC-IV database has received ethical approval from the institutional review boards (IRB) at Beth Israel Deaconess Medical Center and the Massachusetts Institute of Technology Institutional Review Board. Consent was obtained for the original data collection, and informed consent was obtained from all subjects or, if subjects are under 18, from a parent or legal guardian. The ethical review was exempted for the present study by the Institutional Review Board of The Affiliated Hospital of Southwest Medical University.

### Data collection

Demographic and clinical data were extracted from the database. The general parameters included gender, age, body mass index (BMI), race, and comorbidities such as myocardial infarction, heart failure, cerebrovascular disease, diabetes mellitus, etc., Therapy undertaken was also captured, including vasopressor therapy and dialysis procedures. Physiological parameters were also captured including Pplat and Ppeak, Vt, respiratory rate (RR), PEEP, heart rate, blood pressure (systolic, diastolic, mean), body temperature, and SpO₂ (oxygen saturation). Laboratory results included hematology parameters (hematocrit, hemoglobin, white cell count), kidney and metabolic parameters (creatinine, sodium, lactate), and arterial blood gas analysis (pH, PaO₂, PaCO₂). We also presented inspired oxygen fraction (FiO₂), PaO₂/FiO₂ ratio, Berlin severity categories, Vt normalized to predicted body weight, ΔP, static compliance, and ventilatory ratio. Severity of illness was also assessed by the application of sequential organ failure assessment (SOFA), simplified acute physiology score II (SAPS II), and oxford acute severity of Illness score (OASIS). The follow-up durations for mortality endpoints included in-hospital, 28-day, and 90-day all-cause mortality after ICU admission.

### Mechanical power calculations

MP was computed using the simplified equation:


$$MP\;=\text{0}.\text{098}\times \;RR\times Vt\times (Ppeak-\frac{\Delta P}{\text{2}})$$

Where MP is mechanical power (J/min), RR is respiratory rate (breaths/min), Vt is tidal volume (L), Ppeak is peak inspiratory pressure (cmH₂O), and ΔP is driving pressure (cmH₂O). For each ICU stay, we defined MP using the earliest available ventilator recording after ICU admission (within the first 24 hours) [9,14].

### Statistical analysis

Data extraction with subsequent analysis was performed using Navicat 16 to manage databases, PostgreSQL to query data, and Python 3.13 to conduct statistical and computational analysis. Continuous variables were described as mean (standard deviation) and compared across quartiles using analysis of variance (ANOVA). Categorical variables were summarized as a count (percentages) and compared via chi-squared tests. Before undergoing statistical modeling, a check for data completeness for all variables was undertaken. We evaluated the relationship between MP and mortality using Cox proportional hazards regression models to account for time-to-event data. MP was divided into quartiles (Q1–Q4). Missing data (S1 Table) for covariates was solved using multiple imputations by chained equations (MICE). We fit three progressively nested models: Model 1 adjusting for gender, age, BMI, and race; Model 2, with adjustments for SOFA, SAPSII, OASIS; and Model 3, with adjustments for Vt, ΔP, PEEP, and Pplat. Hazard ratios with 95% CIs and p values for Q2 to Q4 compared to Q1 for each model are provided. Model validation was carried out on a representative imputed data set with Schoenfeld residuals to test for proportional hazards assumptions, Harrell’s C-index to assess model discrimination, and risk quintiles for calibration comparisons based on observed and predicted event rates. Logistic regression was employed specifically to generate predicted probabilities for receiver operating characteristic (ROC) curve analysis to compare the discriminative ability of MP against traditional ventilator parameters. To explore potential non-linear links, we considered MP as a continuous variable using restricted cubic splines (RCS) with four degrees of freedom with a choice of median MP as a reference point. Non-linearity testing involved a consideration of the joint significance of spline terms compared to the linear component (p for non-linearity). Kaplan–meier survival analyses were performed across 28-day, and 90-day mortality. We conducted subgroup analyses with logistic regression with interaction terms to investigate to what extent the relationship between MP and mortality end points (28-, and 90-day mortality) differed across clinical subgroups defined by demographic variables and severity scores. In sensitive analysis, we adopted a complete-case approach, excluding any observations with missing values. We also applied inverse probability of treatment weighting (IPTW) according to propensity scores to account for confounders with resulting weighted Cox models for mortality end points with robust variance estimation.

## Result

### Baseline characteristics

Table 1 lists the baseline characteristics for 1,878 Berlin ARDS subjects stratified by MP quartiles. Severe illnesses, indicated by higher MP quartiles, were common, while other variables, such as age, gender, ethnicity, and most comorbid conditions, were generally equally represented. Scoring for severity, represented by SOFA, SAPS II, OASIS, and other scales for respiration, significantly increased with advancing MP, with lower ratios for PaO2/FiO2, lower values for pH, higher PaCO2, and increasing ventilatory ratios. More aggressive ventilator parameters, such as Pplat, ΔP, Vt, RR, Ppeak, and PEEP, significantly increased from Q1 to Q4, while other hemodynamic and laboratory values were generally not significantly different. Survival duration did not significantly vary in any group. Of note, Data completeness was generally high with less than 5% missingness for most key physiological and laboratory variables (e.g., pH, pCO2), whereas derived indices such as BMI and ventilatory ratio showed higher missingness rates (approximately 22–26%) (S2 Table).

**Table 1:** Baseline characteristics of acute respiratory distress syndrome-related patients stratified by mechanical power quartiles (Q1–Q4).

Variable	Overall (n = 1878)	Q1 (n = 470)	Q2 (n = 469)	Q3 (n = 469)	Q4 (n = 470)	p_value
Demographics						
Age	61.59 (15.80)	63.97 (15.76)	64.75 (14.91)	60.61 (15.41)	57.04 (15.96)	< 0.01
Gender						< 0.01
Female	749 (39.9%)	230 (48.9%)	181 (38.6%)	183 (39.0%)	155 (33.0%)	
Male	1129 (60.1%)	240 (51.1%)	288 (61.4%)	286 (61.0%)	315 (67.0%)	
BMI	30.99 (7.83)	28.64 (6.52)	29.84 (6.43)	31.98 (7.66)	33.80 (9.51)	< 0.01
Race						< 0.01
White	1198 (63.8%)	316 (67.2%)	324 (69.1%)	283 (60.3%)	275 (58.5%)	
Others	680 (36.2%)	154 (32.8%)	145 (30.9%)	186 (39.7%)	195 (41.5%)	
Comorbidities						
Cerebrovascular disease	258 (13.7%)	63 (13.4%)	60 (12.8%)	69 (14.7%)	66 (14.0%)	0.85
Chronic pulmonary disease	452 (24.1%)	98 (20.9%)	114 (24.3%)	120 (25.6%)	120 (25.5%)	0.28
Myocardial infarct	296 (15.8%)	81 (17.2%)	73 (15.6%)	76 (16.2%)	66 (14.0%)	0.59
Congestive heart failure	46 (2.5%)	11 (2.3%)	10 (2.1%)	13 (2.8%)	12 (2.6%)	0.93
Peripheral vascular disease	263 (14.0%)	69 (14.7%)	78 (16.6%)	62 (13.2%)	54 (11.5%)	0.13
Dementia	31 (1.7%)	9 (1.9%)	6 (1.3%)	10 (2.1%)	6 (1.3%)	0.64
Rheumatic disease	59 (3.1%)	19 (4.0%)	16 (3.4%)	9 (1.9%)	15 (3.2%)	0.30
Peptic ulcer disease	59 (3.1%)	13 (2.8%)	18 (3.8%)	16 (3.4%)	12 (2.6%)	0.66
Liver disease	375 (20.0%)	67 (14.3%)	86 (18.3%)	100 (21.3%)	122 (26.0%)	< 0.01
Diabetes	534 (28.4%)	117 (24.9%)	129 (27.5%)	147 (31.3%)	141 (30.0%)	0.13
Paraplegia	83 (4.4%)	24 (5.1%)	22 (4.7%)	13 (2.8%)	24 (5.1%)	0.25
Renal disease	280 (14.9%)	70 (14.9%)	74 (15.8%)	68 (14.5%)	68 (14.5%)	0.94
Malignant cancer	208 (11.1%)	44 (9.4%)	52 (11.1%)	57 (12.2%)	55 (11.7%)	0.54
Interventions						
Dialysis active	264 (14.1%)	49 (10.4%)	55 (11.7%)	71 (15.1%)	89 (18.9%)	< 0.01
Vasopressors	1457 (77.6%)	357 (76.0%)	370 (78.9%)	361 (77.0%)	369 (78.5%)	0.68
Severity Scores						
SOFA score	7.72 (4.11)	6.64 (3.91)	7.35 (3.96)	8.02 (3.94)	8.88 (4.30)	< 0.01
Berlin severity						< 0.01
Mild	557 (29.7%)	194 (41.3%)	158 (33.7%)	119 (25.4%)	86 (18.3%)	
Moderate	897 (47.7%)	200 (42.6%)	238 (50.7%)	229 (48.8%)	230 (48.9%)	
Severe	424 (22.6%)	76 (16.2%)	73 (15.6%)	121 (25.8%)	154 (32.8%)	
OASIS	37.73 (7.87)	36.84 (7.70)	36.96 (7.45)	37.77 (7.57)	39.36 (8.47)	< 0.01
SAPSII	45.49 (15.09)	43.11 (14.37)	44.68 (14.52)	46.39 (14.37)	47.79 (16.61)	< 0.01
Laboratory						
Creatinine	1.56 (1.36)	1.32 (1.13)	1.45 (1.26)	1.60 (1.42)	1.87 (1.53)	< 0.01
Hematocrit	28.67 (6.60)	27.44 (5.80)	27.62 (6.15)	28.63 (6.89)	30.99 (6.89)	< 0.01
Hemoglobin	9.53 (2.17)	9.14 (1.95)	9.23 (2.05)	9.55 (2.23)	10.21 (2.27)	< 0.01
Lactate	18.69 (256.33)	18.70 (206.30)	35.38 (438.99)	7.69 (103.40)	13.31 (139.04)	0.41
pH	7.33 (0.12)	7.36 (0.10)	7.34 (0.10)	7.33 (0.12)	7.29 (0.14)	< 0.01
Sodium	140.63 (4.91)	140.35 (4.65)	140.48 (4.83)	140.80 (4.86)	140.89 (5.25)	0.28
WBC	17.12 (10.58)	16.32 (7.75)	16.96 (12.27)	17.76 (11.75)	17.44 (9.92)	0.18
Respiratory/ Ventilator Variables						
Heart rate	87.21 (16.28)	84.96 (14.92)	86.18 (15.19)	87.17 (16.04)	90.51 (18.28)	< 0.01
DBP	59.76 (9.15)	59.20 (8.69)	58.22 (8.53)	60.25 (9.17)	61.35 (9.87)	< 0.01
MBP	76.05 (9.29)	76.27 (8.66)	75.07 (8.60)	76.14 (9.35)	76.71 (10.41)	0.05
SBP	113.17 (13.22)	113.65 (12.84)	113.10 (12.64)	112.97 (12.90)	112.95 (14.47)	0.84
Respiratory rate	18.79 (4.80)	16.09 (3.15)	17.06 (3.22)	18.61 (4.01)	23.39 (4.96)	< 0.01
Temperature	36.87 (0.80)	36.77 (0.66)	36.80 (0.63)	36.87 (0.86)	37.03 (0.98)	< 0.01
Mechanical power	16.53 (7.72)	9.52 (1.70)	12.91 (0.91)	16.67 (1.42)	27.04 (7.71)	< 0.01
Plateau pressure	19.70 (4.85)	16.59 (2.99)	18.17 (3.34)	20.52 (4.36)	23.51 (5.29)	< 0.01
Peak pressure	24.79 (5.83)	19.94 (3.29)	22.66 (3.45)	25.98 (4.19)	30.56 (5.72)	< 0.01
Tidal volume	484.95 (95.12)	438.12 (97.92)	487.21 (73.80)	500.04 (84.17)	514.48 (103.98)	< 0.01
PEEP	6.92 (3.12)	5.39 (1.15)	5.72 (1.46)	6.79 (2.34)	9.77 (4.26)	< 0.01
FiO2	0.82 (0.24)	0.81 (0.25)	0.82 (0.24)	0.80 (0.24)	0.84 (0.22)	0.15
PaCO2	43.46 (11.60)	40.91 (8.70)	41.93 (8.54)	43.53 (11.11)	47.41 (15.50)	< 0.01
SpO2	97.10 (2.78)	97.59 (2.16)	97.59 (1.67)	97.05 (2.73)	96.16 (3.84)	< 0.01
PF ratio	160.77 (67.58)	180.47 (67.92)	170.25 (65.77)	154.69 (65.37)	137.68 (63.53)	< 0.01
SC, L/cmH2O	0.04 (0.02)	0.04 (0.01)	0.04 (0.02)	0.04 (0.03)	0.04 (0.02)	0.96
Ventilatory ratio	1.66 (0.70)	1.27 (0.38)	1.46 (0.40)	1.66 (0.55)	2.28 (0.91)	< 0.01
Vt/PBW	7.71 (1.76)	7.27 (1.78)	7.83 (1.71)	7.87 (1.68)	7.90 (1.81)	< 0.01
Driving pressure	12.78 (3.99)	11.20 (2.85)	12.46 (3.41)	13.73 (4.34)	13.75 (4.57)	< 0.01
Outcomes						
In hospital mortality	431 (23.0%)	74 (15.7%)	83 (17.7%)	118 (25.2%)	156 (33.2%)	< 0.01
Survival time days	135.74 (485.05)	116.09 (437.48)	128.44 (473.86)	142.85 (466.68)	155.58 (554.92)	0.62

BMI, body mass index; SBP, systolic blood pressure; DBP, diastolic blood pressure; MBP, mean blood pressure; SpO2, oxygen saturation; PaCO2, arterial carbon dioxide partial pressure; PF, PaO2/FiO2; PaO2, arterial oxygen partial pressure; WBC, white blood cell; SC, static compliance; Vt/PBW, tidal volume per predicted body weight.

### Mortality risk and model performance

**Fig 2:**
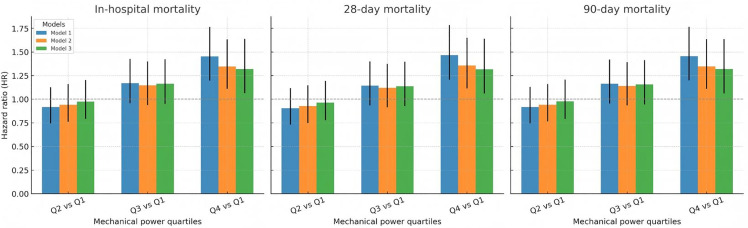
Mortality risk for in-hospital, 28-day, and 90-day mortality across mechanical power quartiles (Q2–Q4).

**Fig 3:**
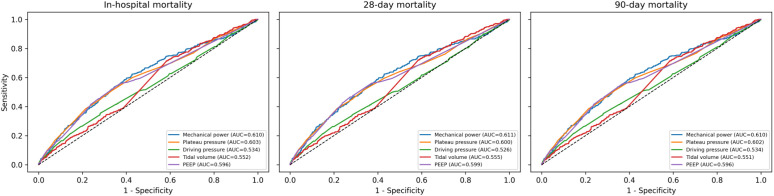
Comparative receiver operating characteristic curves.

At each endpoint, there was a progressively increasing risk of death with increasing quartiles (Fig 2). Relative to Q1, in model 3, adjusted for all covariates, Q4 was associated with a significantly increased risk of death (hazard ratio (HR) 1.32, 95% CI 1.06 to 1.64, p < 0.05), whereas Q2 and Q3 were not significantly different from Q1 (Table 2). Models for Q1-4 did not significantly depart from the proportional hazards assumptions in Cox models, had fair discriminatory capacity (C-index ~0.7), and had good, although slightly heterogeneous, calibration with a slight tendency to overestimate in lowest risk groups (S2 Table). ROC curve analysis showed MP having highest AUC (0.61), significantly different from other variables: Pplat, AUC (0.60), PEEP (0.60), Vt, (0.55), and ΔP (0.53) (Fig 3).

**Table 2:** Association of mechanical power quartiles with mortality outcomes.

Outcome	Quartile	Model 1 HR (95% CI), p	Model 2 HR (95% CI), p	Model 3 HR (95% CI), p
In hospital mortality	Q2	0.92 (0.74–1.13), p = 0.41	0.94 (0.76–1.16), p = 0.56	0.98 (0.79–1.20), p = 0.82
	Q3	1.17 (0.96–1.43), p = 0.12	1.15 (0.94–1.40), p = 0.18	1.16 (0.95–1.42), p = 0.15
	Q4	1.45 (1.20–1.76), p < 0.01	1.35 (1.11–1.63), p < 0.01	1.32 (1.06–1.64), p = 0.01
28-day mortality	Q2	0.90 (0.73–1.12), p = 0.34	0.93 (0.75–1.15), p = 0.48	0.96 (0.78–1.19), p = 0.73
	Q3	1.14 (0.93–1.40), p = 0.20	1.12 (0.92–1.37), p = 0.27	1.14 (0.93–1.40), p = 0.22
	Q4	1.47 (1.21–1.78), p < 0.01	1.36 (1.11–1.65), p < 0.01	1.32 (1.06–1.64), p = 0.01
90-day mortality	Q2	0.92 (0.74–1.13), p = 0.41	0.94 (0.76–1.16), p = 0.57	0.98 (0.79–1.21), p = 0.83
	Q3	1.16 (0.95–1.42),p = 0.14	1.14 (0.93–1.39), p = 0.20	1.16 (0.94–1.41),p = 0.16
	Q4	1.45 (1.20–1.76), p < 0.01	1.35 (1.11–1.63), p < 0.01	1.32 (1.06–1.64), p = 0.01

HR: hazard ratio; CI: confidence interval.

### Restricted cubic spline analysis

**Fig 4:**
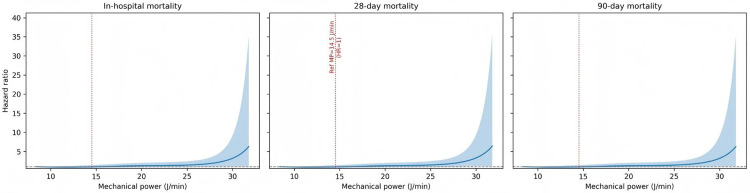
Restricted cubic spline regression analysis of mechanical power with in-hospital, 28-day, and 90-day all-cause mortality.

Across in-hospital, 28-day, and 90-day mortality, RCS analyses under Model 3 showed a broadly monotonic increase in mortality risk with higher mechanical power and no strong statistical evidence of pronounced non-linearity (likelihood ratio tests: LR 6.3, 6.1, and 6.2 with 3 degrees of freedom; p = 0.10, 0.11, and 0.10, respectively) (Fig 4).

### Kaplan–Meier survival curves

**Fig 5:**
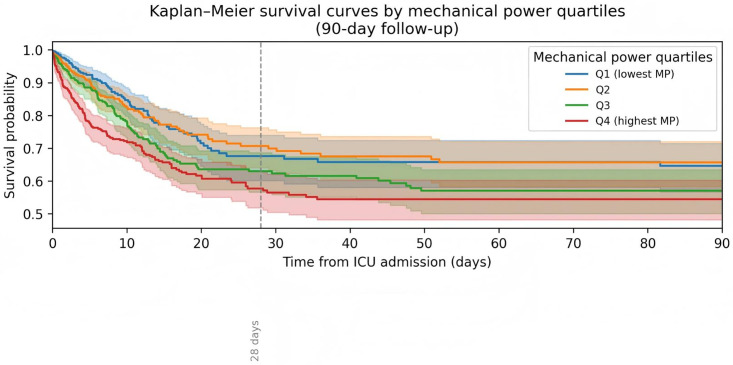
Kaplan-meier survival curves for in-hospital, 28-day, and 90-day all-cause mortality across mechanical power quartiles.

In unadjusted Kaplan–Meier analyses, survival decreased progressively from Q1 to Q4, with Q4 showing the steepest early decline in survival (Fig 5). Notably, while the survival curve for Q1 briefly dropped below Q2 around day 28, it later returned above, and the overall trend of decreasing survival from Q1 to Q4 remained consistent over the 90-day follow-up period.

### Subgroup and sensitivity analysis

In subgroup analyses, the excess risk associated with the highest mechanical power quartile was broadly consistent across sex, age, BMI, race, and severity strata in all subgroups and no strong evidence of effect modification (S3 Table). In sensitivity analyses, Cox models with IPTW adjustment also confirmed the results such that higher mortality risk was evident among high MP subjects compared with low MP subjects, and a complete-case approach yielded effect estimates that were directionally and quantitatively similar to the primary models (S4 and S5 Tables).

## Discussion

Using this retrospective cohort data from ARDS patients in a large ICU database, we observed a consistent association between higher early levels of mechanical power and increased risks of in-hospital, 28-day, and 90-day deaths. Compared with the lowest quartile, there was significantly increased mortality in the highest quartile for mechanical power, with survival curves diverging sharply among quartiles in Kaplan–Meier, analyses. In ROC analyses, MP demonstrated statistically superior but clinically modest discriminative ability (AUC 0.61). While it outperformed individual traditional ventilator variables (e.g., Pplat, ΔP, Vt), its absolute predictive power suggests it functions best as a complementary signal within a multimodal assessment rather than a standalone prognostic marker. These relationships were roughly linear over the clinically observed range, generally upheld in major clinical subgroups, and proved robust to sensitivity analyses, indicating that MP reflects ventilator load’s prognostically informative component in ARDS.

Previous studies have established that ventilatory parameters such as Vt, ΔP, and Pplat correlate significantly with ARDS outcome in patients. We found that the implementation of a lung-protective strategy with smaller Vt resulted in a substantial reduction in mortality compared to standard ventilation strategies, thereby reinforcing the importance of preventing volutrauma as well as barotrauma [15,16]. More currently, ΔP has been established as an important determinant of VILI and is a more accurate predictor of mortality than Vt or Pplat taken individually [5,17]. Observational studies have also confirmed that elevated Pplat are associated with adverse outcome in ARDS [18,19]. As a whole, this collection of evidence highlights the central importance of ventilatory mechanics to outcome prediction and provides a theoretical framework to understand MP as an integrated measure encompassing these disparate elements. An elevated MP worsens VILI through the cumulative effects of volutrauma, barotrauma, and atelectrauma. Extensive observations have determined that elevated MP is an independent predictor of adverse outcome among critically ill patients who receive invasive ventilation [20,21].

Detrimental impacts of higher MP on patient outcomes among patients with ARDS can be explained via VILI. Higher MP increases stress and strain application to lung tissue that is already damaged, amplifying alveolar overdistension and shear forces [22,23]. Accumulation of kinetic energy with each respiratory maneuver results in repeated mechanical disruption of air spaces with resultant accelerated injury to tissues and impairment of repair mechanisms [24,25]. In addition to these effects, excessively high MP has been correlated with higher biotrauma, with resultant incitation of local and systemic inflammation leading to propagation of multi-organ dysfunction [26–28]. This process is critical among ARDS because concomitant low lung compliance increases propagation of injurious stress and strain. In lungs with heterogeneity, even low increases in Vt or airway pressures can be accompanied by excessively high stress and strain with resultant higher risk for VILI [29]. As such, ARDS patients can incur higher adverse effects from increases in MP than their more compliant lung counterparts with critical importance for customization of ventilation strategies to uniquely individual lung mechanics for this individual population [30,31].

Multiple recent assessments with the MIMIC database have again accentuated the predictive value of MP across several critically ill patients. For example, one study showed that higher MP was associated with mortality rates among critically ill patients within the MIMIC-III and eICU observational cohorts [32]. Follow-on studies showed excess MP to correspond with prolonged duration of mechanical ventilation, higher 28-day all-cause mortality in chronic obstructive pulmonary disease patients, and suboptimal weaning outcomes among mechanically ventilated individuals [33–35]. Therefore, rather than serving as a standalone target, MP should be interpreted as an integrative marker of the total ventilatory burden. Its incremental clinical value lies in capturing the cumulative physiological cost of Vt, pressure, and respiratory rate—nuances that isolated variables may miss. This holistic perspective supports individualized management, allowing clinicians to balance trade-offs between specific ventilator settings to minimize the overall energy transfer to the injured lung while maintaining adequate gas exchange.

Our study benefits from a very large real-world cohort with sufficient sample size, employs extensive analytical strategies with Cox models, subgroup and sensitivity analysis, IPTW adjustment for robustness checking, but uniquely incorporates RCS modeling to document a nonlinear relationship between MP and mortality. Nonetheless, our study has some limitations to be taken into consideration. First, it utilized a single-center database, which would restrict our results from being generalized to others. Second, owing to the retrospective design, it is impossible to completely rule out unmeasured confounders. Furthermore, the identification of ARDS relied on physiological criteria without centrally adjudicated review of chest imaging or echocardiography to strictly exclude hydrostatic edema, which risks misclassification bias by potentially including non-ARDS hypoxemic patients. Third, we did not have longitudinal data regarding dynamic ventilatory management changes but only initial or averaged data that would fail to reflect complex management. Fourth, missing data for some variables might lead to bias. Lastly, we were unable to account for potentially relevant interacting interventions such as prone positioning and ECMO. Since these rescue therapies are typically applied to patients with the most severe lung injury and highest ventilatory requirements, their absence from the model may introduce residual confounding in the relationship between MP and mortality.

## Conclusion

Early mechanical power was independently associated with in-hospital, 28-day, and 90-day mortality in patients with ARDS. MP should be viewed as a complementary risk stratification tool that reflects total ventilatory load, warranting further prospective validation to determine its therapeutic utility.

## Supporting information

S1 Table Summary of variables with missing data. (DOCX)

S2. Table Proportional hazards diagnostics, discrimination, and calibration. (DOCX)

S3. Table Subgroup analysis of mortality outcomes. (DOCX)

S4. Table Complete case sensitivity analysis. (DOCX)

S5. Table Inverse probability of treatment weighting sensitivity analysis results. (DOCX)

S1 Data Raw data. (CSV)
