# Supplementary material for: Mechanical power and short-term mortality in critically ill patients with ARDS on mechanical ventilation: Insights from the MIMIC-IV database
Source: PLoS One. 2026 Feb 2;21(2):e0341923. doi: 10.1371/journal.pone.0341923 (PMC12863555; doi:10.1371/journal.pone.0341923)
Supplement: S1 Table — (DOCX) [file pone.0341923.s001.docx]

**S1 Table. Summary of variables with missing data**

| **Variable** | **n_missing** | **prop_missing** |
| --- | --- | --- |
| Ventilatory_ratio | 479 | 25.51% |
| BMI | 417 | 22.20% |
| Vt_pbw_ml_per_kg | 417 | 22.20% |
| Temperature | 170 | 9.05% |
| Lactate | 98 | 5.22% |
| pH | 78 | 4.15% |
| PaCO2 | 78 | 4.15% |
| FiO2 | 57 | 3.03% |
| Static_compliance_l_per_cmH2O | 5 | 027% |
| Hematocrit | 1 | 0.05% |
| Sodium | 1 | 0.05% |
| Hemoglobin | 1 | 0.05% |
