# Supplementary material for: Mechanical power and short-term mortality in critically ill patients with ARDS on mechanical ventilation: Insights from the MIMIC-IV database
Source: PLoS One. 2026 Feb 2;21(2):e0341923. doi: 10.1371/journal.pone.0341923 (PMC12863555; doi:10.1371/journal.pone.0341923)
Supplement: S2. Table — (DOCX) [file pone.0341923.s002.docx]

**S2 Table. Proportional hazards diagnostics, discrimination, and calibration**

| **Endpoint** | **model** | **n** | **t0_calibration** | **c_index** | **pH_p_mpQ2** | **pH_p_mpQ3** | **pH_p_mpQ4** | **pH_p_min_all** | **mean_pred_risk_all** | **obs_event_rate_all** | **low_q_n** | **low_q_pred** | **low_q_obs** | **high_q_n** | **high_q_pred** | **high_q_obs** |
| --- | --- | --- | --- | --- | --- | --- | --- | --- | --- | --- | --- | --- | --- | --- | --- | --- |
| D28 | M1 | 1878 | 28 | 0.61 | 0.55 | 0.66 | 0.00 | 0.00 | 0.36 | 0.22 | 376 | 0.28 | 0.15 | 376 | 0.44 | 0.36 |
| D28 | M2 | 1878 | 28 | 0.73 | 0.50 | 0.68 | 0.02 | 0.02 | 0.32 | 0.22 | 376 | 0.15 | 0.05 | 376 | 0.59 | 0.49 |
| D28 | M3 | 1878 | 28 | 0.73 | 0.53 | 0.81 | 0.25 | 0.03 | 0.32 | 0.22 | 376 | 0.14 | 0.06 | 376 | 0.59 | 0.54 |
| D90 | M1 | 1878 | 90 | 0.61 | 0.72 | 0.82 | 0.00 | 0.00 | 0.39 | 0.23 | 376 | 0.31 | 0.15 | 376 | 0.49 | 0.37 |
| D90 | M2 | 1878 | 90 | 0.73 | 0.68 | 0.83 | 0.02 | 0.02 | 0.36 | 0.23 | 376 | 0.17 | 0.06 | 376 | 0.64 | 0.52 |
| D90 | M3 | 1878 | 90 | 0.73 | 0.67 | 0.94 | 0.24 | 0.05 | 0.36 | 0.23 | 376 | 0.16 | 0.06 | 376 | 0.65 | 0.56 |
| Hosp | M1 | 1878 | 90 | 0.61 | 0.71 | 0.89 | 0.00 | 0.00 | 0.39 | 0.23 | 376 | 0.31 | 0.16 | 376 | 0.49 | 0.38 |
| Hosp | M2 | 1878 | 90 | 0.73 | 0.66 | 0.90 | 0.01 | 0.01 | 0.36 | 0.23 | 376 | 0.17 | 0.06 | 376 | 0.64 | 0.52 |
| Hosp | M3 | 1878 | 90 | 0.73 | 0.66 | 0.99 | 0.24 | 0.04 | 0.36 | 0.23 | 376 | 0.16 | 0.06 | 376 | 0.65 | 0.56 |
| Abbreviations: Hosp, in-hospital mortality; D28, 28-day mortality; D90, 90-day mortality; M1–M3, nested adjustment models; C-index, Harrell’s concordance index; ph_p_mpQ2–4, p-values from Schoenfeld residual tests for mechanical power quartiles Q2–Q4; ph_p_min_all, minimum p-value across all covariates; mean_pred_risk_all, average predicted risk at t0; obs_event_rate_all, observed event rate at t0; low_q_pred/obs and high_q_pred/obs, mean predicted risk and observed event rate in the lowest and highest quintiles of predicted risk, respectively. | | | | | | | | | | | | | | | | |
