# Supplementary material for: Mechanical power and short-term mortality in critically ill patients with ARDS on mechanical ventilation: Insights from the MIMIC-IV database
Source: PLoS One. 2026 Feb 2;21(2):e0341923. doi: 10.1371/journal.pone.0341923 (PMC12863555; doi:10.1371/journal.pone.0341923)
Supplement: S3. Table — (DOCX) [file pone.0341923.s003.docx]

**S3. Table. Subgroup analysis of mortality outcomes**

| **Outcome** | **OR 95% CI** | **p-value** | **p-value for interaction** |
| --- | --- | --- | --- |
| In-Hospital | | | |
| Gender | | | < 0.01 |
| Female | 1.41 (0.96 - 2.07) | 0.08 |  |
| Male | 2.76 (2.04 - 3.02) | < 0.01 |  |
| Age | | | 0.89 |
| < 65 | 2.10 (1.54 - 2.84) | < 0.01 |  |
| ≥ 65 | 2.17 (1.49 - 3.16) | < 0.01 |  |
| BMI | | | 0.17 |
| ≥ 30 | 1.72 (1.14 - 2.57) | < 0.01 |  |
| < 30 | 2.44 (1.83 - 3.27) | < 0.01 |  |
| Race |  | | 0.21 |
| Others | 1.68 (1.17 - 2.42) | < 0.01 |  |
| White | 2.28 (1.68 - 3.10) | < 0.01 |  |
| SOFA Score | | | 0.15 |
| ≥ 8 | 1.92 (1.44 - 2.56) | 0.35 |  |
| < 8 | 1.26 (0.78 - 2.04) | < 0.01 |  |
| SAPSII | | | 0.84 |
| ≥ 45 | 1.86 (1.38 - 2.51) | < 0.01 |  |
| < 45 | 1.97 (1.28 - 3.03) | < 0.01 |  |
| OASIS |  |  | 0.75 |
| ≥ 38 | 1.90 (1.42 - 2.55) | < 0.01 |  |
| < 38 | 1.75 (1.14 - 2.67) | 0.01 |  |
| 28-Days | | | |
| Gender | | | 0.01 |
| Female | 1.50 (1.01 - 2.21) | 0.04 |  |
| Male | 2.81 (2.07 - 3.81) | < 0.01 |  |
| Age |  |  | 0.88 |
| ≥ 65 | 2.13 (1.45 - 3.12) | < 0.01 |  |
| < 65 | 2.21 (1.63 - 3.01) | < 0.01 |  |
| BMI | | | 0.22 |
| ≥ 30 | 1.82 (1.20 - 2.76) | < 0.01 |  |
| < 30 | 2.51 (1.87 - 3.36) | < 0.01 |  |
| Race | | | 0.22 |
| Others | 1.75 (1.21 - 2.52) | < 0.01 |  |
| White | 2.36 (1.73 - 3.22) | < 0.01 |  |
| SOFA Score | | | 0.22 |
| ≥ 8 | 1.96 (1.46 - 2.62) | < 0.01 |  |
| < 8 | 1.37 (0.84 - 2.23) | 0.21 |  |
| SAPSII | | | 0.94 |
| ≥ 45 | 1.96 (1.45 - 2.64) | < 0.01 |  |
| < 45 | 1.99 (1.29 - 3.09) | < 0.01 |  |
| OASIS |  |  | 0.92 |
| ≥ 38 | 1.93 (1.44 - 2.60) | < 0.01 |  |
| < 38 | 1.88 (1.22 - 2.90) | < 0.01 |  |
| 90-Days | | | |
| Gender | | | 0.01 |
| Female | 1.41 (0.96 - 2.07) | 0.08 |  |
| Male | 2.78 (2.06 - 3.76) | < 0.01 |  |
| Age | | | 0.86 |
| ≥ 65 | 2.19 (1.50 - 3.19) | < 0.01 |  |
| < 65 | 2.10 (1.54 - 2.84) | < 0.01 |  |
| BMI |  |  | 0.18 |
| ≥ 30 | 1.74 (1.16 - 2.62) | 0.01 |  |
| < 30 | 2.44 (1.83 - 3.27) | < 0.01 |  |
| Race | | | 0.02 |
| Others | 1.68 (1.17 - 2.42) | < 0.01 |  |
| White | 2.30 (1.69 - 3.12) | < 0.01 |  |
| SOFA Score | | | 0.14 |
| ≥ 8 | 1.93 (1.45 - 2.58) | < 0.01 |  |
| < 8 | 1.26 (0.77 - 2.04) | 0.35 |  |
| SAPSII |  |  | 0.86 |
| ≥ 45 | 1.88 (1.39 - 2.53) | < 0.01 |  |
| < 45 | 1.97 (1.28 - 3.03) | < 0.01 |  |
| OASIS |  |  | 0.79 |
| ≥ 38 | 1.90 (1.42 - 2.55) | < 0.01 |  |
| < 38 | 1.77 (1.16 - 2.71) | 0.01 |  |
| BMI, body mass index; SOFA, sequential organ failure assessment; SAPSII, simplified acute physiology score II; OASIS: oxford acute severity of illness score; OR: odds ratio; CI: confidence interval. | | | |
