# Supplementary material for: Mechanical power and short-term mortality in critically ill patients with ARDS on mechanical ventilation: Insights from the MIMIC-IV database
Source: PLoS One. 2026 Feb 2;21(2):e0341923. doi: 10.1371/journal.pone.0341923 (PMC12863555; doi:10.1371/journal.pone.0341923)
Supplement: S4. Table — (DOCX) [file pone.0341923.s004.docx]

**S4. Table. Complete case sensitivity analysis**

| **Outcome** | **Quartile** | **Model 1**  **HR (95% CI), p** | **Model 2**  **HR (95% CI), p** | **Model 3**  **HR (95% CI), p** |
| --- | --- | --- | --- | --- |
|  |  |  |  |  |
| In hospital mortality | Q2 | 0.92 (0.72–1.17), p = 0.47 | 0.94 (0.74–1.20), p = 0.64 | 0..98 (0.77–1.25), p = 0.88 |
|  | Q3 | 1.21 (0.96–1.53), p = 0.10 | 1.21 (0.96–1.53), p = 0.11 | 1.22 (0.96–1.55), p = 0.10 |
|  | Q4 | 1.45 (1.15–1.83), p < 0.01 | 1.36 (1.08–1.71), p = 0.01 | 1.32 (1.02–1.70), p = 0.04 |
| 28-days mortality | Q2 | 0.89 (0.69–1.14), p = 0.35 | 0.92 (0.71–1.17), p = 0.49 | 0.95 (0.74–1.23), p =0.71 |
|  | Q3 | 1.19 (0.94–1.51), p = 0.14 | 1.19 (0.94–1.51), p = 0.15 | 1.21 (0.59–1.53), p = 0.13 |
|  | Q4 | 1.46 (1.16–1.84), p < 0.01 | 1.37 (1.08–1.73), p = 0.01 | 1.32 (1.02–1.71), p = 0.04 |
| 90-days mortality | Q2 | 0.92 (0.72–1.17), p = 0.48 | 0.95 (0.74–1.21), p = 0.65 | 0.98 (0.77–1.26), p = 0.89 |
|  | Q3 | 1.20 (0.95–1.52), p = 0.12 | 1.20 (0.95–1.52), p = 0.13 | 1.21 (0.95–1.53), p = 0.11 |
|  | Q4 | 1.45 (1.15–1.83), p < 0.01 | 1.36 (1.08–1.71), p = 0.01 | 1.32 (1.02–1.70), p = 0.04 |
| HR: hazard ratio; CI: confidence interval. | | | | |
