# Supplementary material for: Mechanical power and short-term mortality in critically ill patients with ARDS on mechanical ventilation: Insights from the MIMIC-IV database
Source: PLoS One. 2026 Feb 2;21(2):e0341923. doi: 10.1371/journal.pone.0341923 (PMC12863555; doi:10.1371/journal.pone.0341923)
Supplement: S5. Table — (DOCX) [file pone.0341923.s005.docx]

**S5 Table. Inverse probability of treatment weighting sensitivity analysis results**

| **Outcome** | **IPTW-adjusted Cox models, HR (95% CI)** | **p-value** |
| --- | --- | --- |
| In-hospital mortality | 1.54 (1.18 - 2.01) | p < 0.01 |
| 28-days mortality | 1.57 (1.20 - 2.05) | p < 0.01 |
| 90-days mortality | 1.54 (1.18 - 2.01) | p < 0.01 |
| IPTW, inverse probability of treatment weighting; HR, hazard ratio; CI, confidence interval. | | |
